# Decoding mutational signatures in breast cancer: Insights from a multi-cohort study

**DOI:** 10.1016/j.tranon.2025.102315

**Published:** 2025-02-04

**Authors:** Margaux Betz, Andréa Witz, Julie Dardare, Cassandra Michel, Vincent Massard, Romain Boidot, Pauline Gilson, Jean-Louis Merlin, Alexandre Harlé

**Affiliations:** a Service de Biopathologie, Institut de Cancérologie de Lorraine, Université de Lorraine, CNRS UMR 7039 CRAN, 54519 Vandœuvre-lès-Nancy, France; b Service de Biopathologie, Institut de Cancérologie de Lorraine, 54519 Vandœuvre-lès-Nancy, France; c Département d'Oncologie Médicale, Institut de cancérologie de Lorraine, 54519 Vandoeuvre-lès-Nancy, France; d Research Platform in Biological Oncology, Center GF Leclerc, Dijon, France

**Keywords:** Mutational signature, Breast cancer, Next generation sequencing, Genomic database

## Abstract

**Purpose:**

Diagnosis and treatment decisions of hormonal breast cancers (BC) are now guided by genomic mutations determination, combined into mutational signatures, and provide insight into the patients’ genomic landscape. This work aims to compare genomic data and signatures extracted from tissue samples collected in the CICLADES study to existing cohorts. Ultimately, the goal is to prove the accuracy of smaller cohorts and provide new relevant data.

**Materials and methods:**

DNA from patients of the CICLADES cohort was extracted, sequenced, and custom filtering was applied to the resulting files. Genomic data was pulled from 6 BC cohorts available on cBioPortal.com. In total, 2303 samples were analyzed. Mutational signatures were extracted and matched to known signatures of the Catalogue of Somatic Mutations in Cancer (COSMIC). Tumor Mutation Burden (TMB) and hypermutation were estimated and compared between samples.

**Results:**

*PIK3CA* and *TP53* represented the two genes highly mutated across all cohorts. TMB was similar between the CICLADES and CBSM groups, however the MSKCC population showed a significantly higher TMB than both. Nine signatures were extracted, with recurring Single Base Substitutions (SBS) signatures like SBS1, SBS2 and SBS5. The presence of APOBEC-specific signatures was concordant with cohorts presenting APOBEC enrichment. The mean number of mutations was significantly higher in enriched samples for each analyzed cohort.

**Conclusion:**

The use of comprehensive genomic profiling provided accurate evaluation of the TMB and extraction of signatures consistent with published literature. The genomic analysis of the tissue samples of the CICLADES cohort brings new and relevant data, comparable to results found in bigger cohorts.

## Highlights

• *PIK3CA* and *TP53* are the most frequently mutated genes among all studied cohorts.
• Tumor mutation burden can be accurately estimated for smaller cohorts.
• Mutational signatures are dependent on the sample type and sequencing method.
• APOBEC-associated signatures are correlated with APOBEC enriched cohorts.

## Introduction

In 2022, breast cancer (BC) was the second most diagnosed cancer across the globe after lung cancer with over 2.30 million cases found worldwide [1] and ranks first in terms of incidence and mortality in more than one hundred countries [2]. BCs can be divided into different subgroups depending on their histological and molecular characteristics.

Three phenotypes can be used to qualify BCs, Estrogen Receptor (ER), Progesterone Receptor (PR) and Human Epidermal Growth Factor Receptor 2 (HER2) [3]. Estrogen and progesterone receptors are expressed in a majority of BCs, condition responsiveness to hormonal therapy and correspond to ER-positive and PR-positive BCs, respectively [4]. Moreover, in approximately 15 % of BCs, the gene encoding HER2 is amplified, leading to an overexpression of this growth factor. These BCs are called HER2-positive BCs [5]. ER-positive and PR-positive BCs are considered as hormonal BCs, allowing patients to benefit from endocrine therapy, in addition to chemotherapy and targeted therapy [6]. The rise of genome-wide sequencing has allowed for a deeper understanding of the underlying mechanisms of cancer biology through genomic mutations, with whole-genome sequencing (WGS), whole-exome sequencing (WES) and targeted sequencing being the three main techniques used today.

The somatic mutations found through these methods can be associated with defects in the DNA replication machinery, the DNA repair system, exposure to mutagens and many other factors. The combinations of mutation types and mutational processes are defined as mutational signatures. In 2013, more than 4 million mutations were combined into 20 distinct mutational signatures [7]. In 2020, those signatures were perfected with additional sequencing data and stored in the Catalogue of Somatic Mutations in Cancer (COSMIC) as the reference signatures. Overall, 26 signatures extracted from were associated with BC [8]. They are sorted into 3 groups of signatures, depending on the number of nucleotides evaluated. Within the first group, signatures are composed of a combination of 96 possible mutations, based on Single Base Substitutions (SBS), and include half of the overall 26 associated signatures. Next, Doublet Base Substitutions (DBS) are considered and amount to 78 strand-agnostic substitutions possible. Five DBS signatures were associated with breast cancer. Finally, the remaining associated 8 signatures belong to the small Insertion and Deletion (ID) group, which is characterized by addition or loss of fragments of DNA between 1 and 50 base pairs.

The CICLADES study (NCT03318263) gathered genomic data from patients who were diagnosed with advanced ER+/HER2- metastatic BC (mBC), treated with aromatase inhibitors (AI) and CDK4/6 inhibitors. The purpose of this article is to compare extracted signatures and relevant genomic information from the newly analyzed CICLADES cohort to the available public data sets found in the cBioPortal database.

## Methods

### Population characteristics

The first 19 patients of the CICLADES (NCT03318263) trial were selected out of the 146 patients included in the cohort. All these patients gave written informed consent for ancillary molecular analyses. These patients presented with metastatic or advanced breast cancer and were treated with both aromatase inhibitors and CDK4/6 inhibitors. The FFPE (Formalin Fixed Paraffin Embedded) samples were removed from archival, and the matching slides were analyzed by a pathologist to determine the area with the greatest percentage of tumor cells. In total, 8 samples were from mastectomies, 4 from bone metastasis, 3 from lymph nodes, 1 from skin metastasis, 1 from nephrectomy and finally, 1 from thoracentesis.

### DNA extraction and quality control

Macrodissection of FPPE samples was performed to obtain five section of 10 µm for DNA extraction. The tumor DNA was extracted using the AllPrep FFPE DNA RNA (Qiagen, Hilden, Germany) kit, with a volume of elution of 30 µL for RNA, and a volume ranging from 50 to 100 µL for DNA, depending on the origin of the FFPE sample. Both RNA and DNA concentration were measured using the Qubit® 3.0 Fluorometer (Invitrogen, Carlsbad, CA, USA).

### Sequencing

NGS libraries were prepared using a custom SureSelect XT HS2 CGP Panel (Agilent Technologies, Santa Clara, California, USA) and targeted exome capture was performed according to the manufacturer's recommendation. A total of 519 full exon coding genes were targeted with a genomic coverage of 2.15 Mb.

Paired-end sequencing was done on a NextSeq550 platform (Illumina, San Diego, USA) using high output 2 × 150 bp reads.

### Data analysis of CICLADES samples

Briefly, the FASTQ files were aligned to the reference genome (GRCh37) with the BWA-MEM software followed by variant calling with the GATK pipeline, in order to obtain BAM files and to generate VCF files, respectively. Annotation of the variants within the VCF files was performed using Ensembl VEP and the resulting file was converted to a MAF file through the vcf2maf pipeline. The final MAF files were filtered and analyzed with the R package Maftools (v2.14.0, https://github.com/PoisonAlien/maftools). For gene mutation analysis, only pathogenic or likely pathogenic variants with a variant allele frequency (VAF) between 2 and 95 % and a minimum of 59 mutated reads were retained. For mutation signature extraction, only variants with a VAF ≤ 2 % and ≥ 95 % were filtered out.

Curated variants, including Single Nucleotide Variants (SNVs) and insertions and deletions (indels), provided by the existing cohorts were left untouched for the genomic alteration analysis. Only variants within any of three categories of clinical significance were selected, namely uncertain significance, likely pathogenic and pathogenic, as described by the American College of Medical Genetics and Genomics (ACMG) [9]. Separate files were generated for each analysis. The Tumor Mutation Burden (TMB) was calculated using the Maftools package, and the size of the sequencing panel was adjusted for each analysis. In cases of pooled cohorts, the TMB of each sub-cohort was calculated with their respective panel size and the results were pooled together.

### Data source and processing

Clinical and genomic data were downloaded from cBioPortal (https://www.cbioportal.org). In total, sequencing results from matched tumor and normal DNA samples from 6 separate cohorts were downloaded. Due to the limited number of individual samples available, 4 cohorts were merged into one group : the Clinical Proteomic Tumor Analysis Consortium (CPTAC) cohort(10), the Breast Invasive Carcinoma cohort from the Broad Institute (BROAD) [11], the Breast Invasive Carcinoma cohort from the Sanger Institute (SANGER) [12] and finally the cohort from the Metastatic Breast Cancer (MBC) Project [13]. The Breast Cancer cohort from the Memorial Sloan Kettering Cancer Center (MSKCC) [14] and the Molecular Taxonomy of Breast Cancer International Consortium (METABRIC) [15] cohort were analyzed separately.

Each group or cohort were filtered out according to clinical information on the hormonal status of the patients enrolled. For the four-cohort group, hereafter called CBSM, only ER+/HER2- patients were included. For the MSKCC cohort, only HR+/HER2- patients were selected because the distinction between ER+ and PR+ patients was not available.

Finally, for the METABRIC cohort, only the ER+/HER2 neutral patients were selected. If more than one tumor sample was sequenced for a patient due to sequential sampling, only the first available sample was selected. The total number of samples analyzed, including the methods of sequencing are listed in Table 1.

**Table 1:** Overall view of the analyzed cohorts.

Group	Cohort	Overall number of patients	Hormonal BC	Number of patients selected	Type of sequencing
CICLADES	CICLADES	19	19	19	CGP
CBSM	CPTAC	122	63	63	WES
	BROAD	103	37	37	WES
	SANGER	100	54	54	WES
	MBC	301	144	37	WES
METABRIC	METABRIC	2509	1143	830	Targeted
MSKCC	MSKCC	1756	1365	1263	Targeted

All groups and their corresponding cohorts were listed, with the initial number of patients in each cohort, the final number of patients selected and the method of sequencing used for the samples. CGP = Comprehensive Genomic Profiling, WES = Whole Exome Sequencing.

### Signature extraction and matching

Signatures were extracted for each cohort with the package sigminer (v2.3.0, https://github.com/ShixiangWang/sigminer) using a non-negative matrix factorization (NMF) algorithm. The resulting signatures were fitted to the COSMIC reference database (v3.4) [16] with applied cosine similarity analysis.

### Statistical analysis

The TMB and the APOBEC (Apolipoprotein B mRNA editing enzyme catalytic polypeptides) enrichment of the cohorts were studied with the non-parametric Mann-Whitney test on GraphPad Prism version 9.5.1 (San Diego, California USA, www.graphpad.com)

## Results

### Genomic alterations

**Fig. 1:**
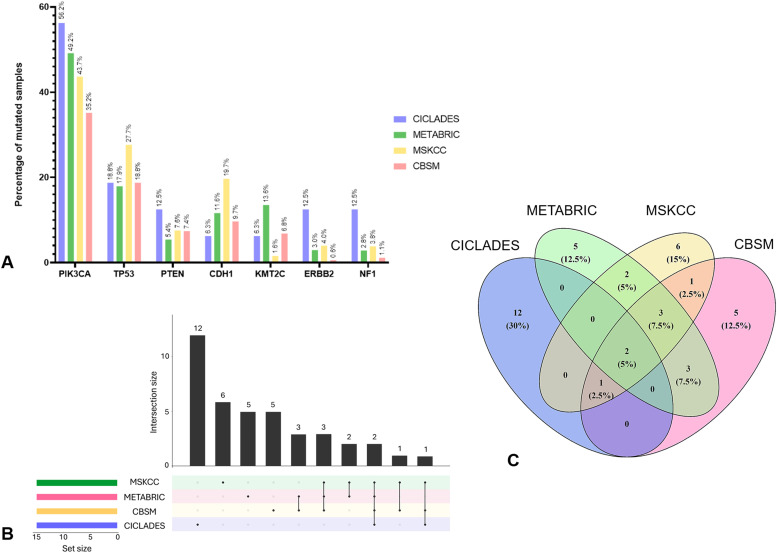
A. Listing of 7 genes of interest and their percentage of mutated samples for each group analyzed. B. and C. Dual representation of the commonly mutated genes between the groups. B. Upset plot generated with the top 15 mutated genes of each group, created with the UpsetR package [26] (v1.4.0). The top plot shows the size of each intersection, while the bottom blot shows from which groups the intersections are occurring C. Venn diagram generated with the same top 15 mutated genes of each group, created with the VennDiagram package [27] (v1.7.3). The number of genes in each intersection is listed with its corresponding percentage.

Altogether, 2303 samples were sequenced, which allowed us to visualize the full genomic landscape of these breast cancer patients. Mutated genes are common between cohorts, but their prevalence vary between populations. Seven relevant genes mutated in all cohorts and their mutation frequency are reported in Fig. 1.A. Among the four groups, *PIK3CA* was the most mutated gene, with a mean mutation rate of 46.05 ± 8.90 % (range, 35.16 % to 56.25 %; *n* = 4 groups). *TP53, PTEN* and *CDH1* had mean mutation rates of 20.77 ± 4.60 % (range, 18.75 % to 27.66 %; *n* = 4 groups), 8.21 ± 3.01 % (range, 5.42 % to 12.50 %; *n* = 4 groups) and 11.80 ± 5.70 % (range, 6.25 % to 19.68 %; *n* = 4 groups), respectively. The mean mutation rates of *KMT2C, ERBB2* and *NF1* for the 4 groups were 7.05 ± 4.92 % (range 1.58 % to 13.55 %), 5.00 ± 5.19 % (range, 0.57 % to 12.50 %) and 5.07 ± 5.07 % (range, 1.14 % to 12.50 %)*,* respectively. As indicated in Fig. 1.B and C, two genes are shared by all four groups. They were identified as *TP53* and *PIK3CA* (*n* = 2; 5 %), and *PTEN* represent the only other gene shared by CICLADES cohort and other populations (MSKCC and CBSM, *n* = 1; 2.5 %). The three remaining populations (MSKCC, CBSM and METABRIC) have in common three genes: *MUC16, GATA3* and *MAP3K1* (*n* = 3; 7.5 %).

Next, the CBSM and METABRIC population have the *KMT2C, CDH1* and *AHNAK* genes in their top 15 mutated genes (*n* = 3; 7.5 %), while the *CBFB* and *TBX3* are shared exclusively by the METABRIC and MSKCC cohorts (*n* = 2; 5 %). Finally, the single gene shared between the CBSM and MSKCC populations as shown in Fig. 1.B and C is the *AKT1* gene (*n* = 1; 2.5 %).

The remaining top 15 mutated genes are exclusive to each cohort (CICLADES: 12 genes; METABRIC: 5 genes; MSKCC: 6 genes; CBSM: 5 genes) accounting to a total of 40 different genes across populations*.*

**Fig. 2:**
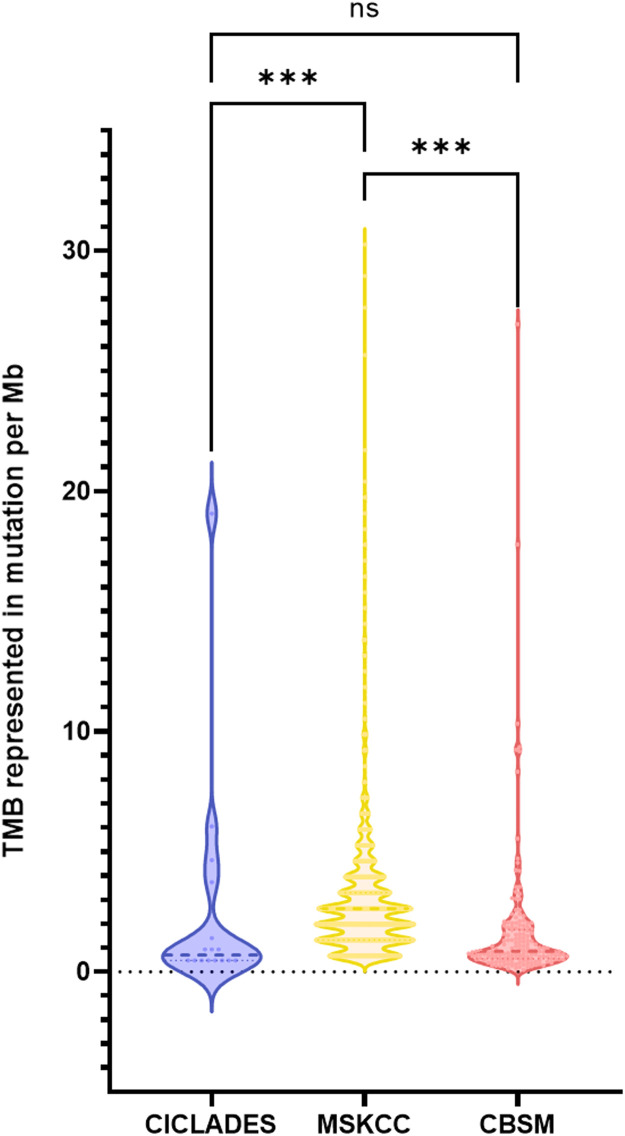
Violin plots depict the distribution of the TMB within the analyzed samples. Data is represented in number of mutations per Mega base (Mb). ***: *P* < 0.001 and ns not significant (Mann-Whitney u-test).

To further assess the number of acquired mutations within the genome of the studied populations, TMB was calculated for three of the four groups. Indeed, the METABRIC cohort was excluded from this analysis as less than 300 genes were included in the sequencing panel. The median TMB was 0.69 mut/Mb (*n* = 16 samples), 0.84 mut/Mb (*n* = 1203 samples) and 2.63 mut/Mb (*n* = 167 samples) for the CICLADES, CBSM and MSKCC groups, respectively (Fig. 2). There is no significant difference in TMB between the CICLADES and CBSM groups (*P* = 0.718). However, the TMB is significantly lower in both the CBSM (*P* < 0.001) and the CICLADES (*P* < 0.001) cohorts compared to the MSKCC cohort. In total, 39 patients had a TMB considered high (≥ 10 mut/Mb), which represented approximately 2.81 % of all samples (39 out of 1386 samples).

### Mutational signatures

In total, nine distinct COSMIC validated signatures were fitted against the extracted mutation patterns. In the CICLADES population, three COSMIC signatures were a match for four different SBS patterns: SBS5, SBS30 and SBS6, as shown in Fig 2.B.

**Fig. 3:**
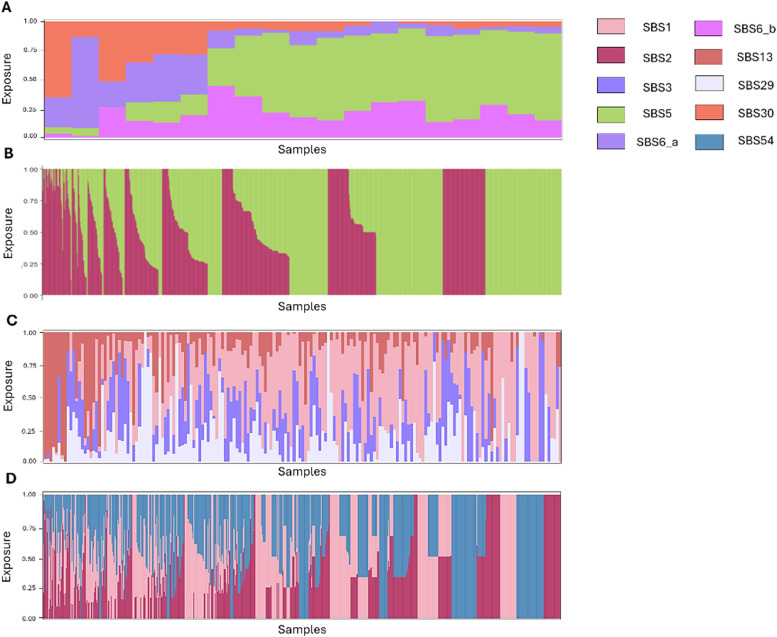
Representation of the exposure of each signature within the analyzed groups. The contribution of each signature is fractioned by normalizing its exposure to the total exposure of each sample, represented by one stack. A. Exposure of the 19 analyzed samples of the CICLADES group. B. Exposure of the 1128 analyzed samples of the MSKCC group. C. Exposure of the 762 analyzed samples of the CBSM group. D. Exposure of the 176 analyzed samples of the METABRIC group.

The clock-like signature SBS5, associated with aging, is the major contributing signature with 63.15 % of samples enriched (12 out 19 analyzed samples, Fig. 3.A), followed by SBS30 in 15.78 % of samples (3 out of 19 analyzed samples), associated with a deficiency of the base excision repair (BER) system.

The signature SBS6 was found in two different groups of samples (SBS6_a and SBS6_b in Fig. 3.A), which were pooled together and amount to 21.05 % of the samples (4 out of 19 analyzed samples), linked with defective DNA mismatch repair (MMR).

In the MSKCC cohort, SBS5 was also the most represented signature at 56.82 % (641 out of 1128 analyzed samples), the rest of the samples showing a majority of SBS2 patterns (43.18 %; 487 out of 1128 analyzed samples), caused by Apolipoprotein B mRNA editing enzyme catalytic polypeptides (APOBEC) activity (Fig. 3.B). APOBEC proteins are cytidine deaminases generating mainly C > T transitions and C > G transversions.

APOBEC proteins are the main cause of signatures SBS2 and SBS13. As shown in Fig. 3.D, SBS2 is enriched in the METABRIC cohort, representing 22.75 % of the patients analyzed (172 out of 762 analyzed samples, Fig. 3.D). Two other mutational signatures are found in the METABRIC cohort, SBS1 and SBS54. SBS1 is a clock-like signature, similar to SBS5, but finds its etiology associated with 5 - methylcytosine enzymatic deamination, potentially caused by FFPE samples handling. It is expressed in 36.48 % of the METABRIC population (278 out of 762 analyzed samples). The third expressed signature is SBS54, its etiology is unknown but is supposedly due to sequencing artefacts or contaminating germline variants and is enriched in 40.94 % of the METABRIC samples (312 out of 762 analyzed samples).

The other signature associated with APOBEC, SBS13, is enriched in 18.75 % (33 out of 176 analyzed samples) of the patients in the CBSM group (Fig. 3.C). SBS1 was also found to be enriched in this cohort, expressed in 42.04 % of the population (74 out of 176 analyzed samples). Finally, two other signatures were extracted in the CBSM cohort: SBS3 and SBS29.

The mutational signature SBS3 is found to be caused by a defective homologous recombination DNA (HRD) system and is enriched in 18.75 % of the patients (33 out of 176 analyzed samples).

Surprisingly, the last signature observed in this cohort is SBS29 which is associated with tobacco chewing, this pattern is found in 36 patients out of the 176 analyzed samples (20.45 %).

### Hypermutation

After unveiling patterns of cytidine deaminase activity in the form of SBS2 and SBS13, we investigated further the presence of APOBEC activity. The CICLADES samples make up the smallest population and none of them are enriched in APOBEC.

However, APOBEC enriched samples represent 23.66 % (159 out of 672 samples) of all METABRIC analyzed samples. The mean number of mutations per samples in the enriched population was significantly higher than in the non-enriched population (5.89 mutations *versus* 4.47 mutations, *P* < 0.001). Similarly, the MSKCC and CBSM enriched APOBEC samples presented a higher mean number of mutations than their non-enriched counterparts (6.49 mutations *versus* 3.82 mutations, *P* < 0.001 and 117.7 *versus* 32.82 mutations, *P* < 0.001, respectively).

**Fig. 4:**
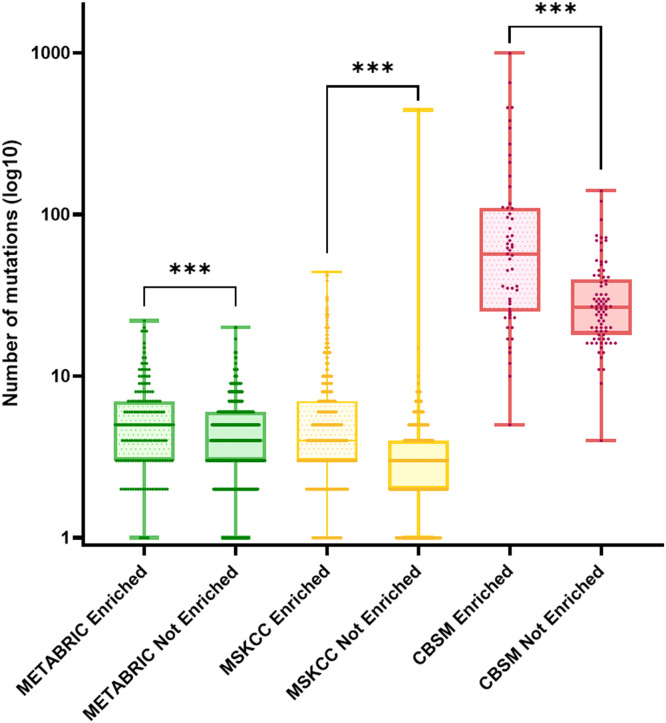
Box plot representation of the distribution of number of mutations between APOBEC enriched and APOBEC not-enriched samples. Data is represented in log10 number of mutations per sample, ***: *P* < 0.001 (Mann-Whitney u-test).

The enriched MSKCC samples represent 33.66 % (350 out of 906 samples) of all MSKCC analyzed samples, while the enriched CBSM samples add up to 40.14 % (55 out of 137 samples) of the overall population (Fig. 4).

## Discussion

Genomic testing has become a standard in cancer diagnostics and treatment choice with the development of personalized medicine. While the method of sequencing used differs between the samples presented previously, the origin and type of fixation of the tissue sample is the first bias among cohorts. For the CICLADES cohort, samples were biopsied from various locations, such as breast mastectomies, lymph nodes, skin or bone metastasis for example, but they were fixed with formalin and embedded in paraffin. Similarly, FFPE samples were used for the MSKCC [14] and MBC [13] populations, while fresh frozen samples were used for DNA extraction in the CPTAC [10], METABRIC [15] and BROAD [11] cohorts. Origins of the tissue also vary between cohorts, lumpectomy and mastectomy were mostly used in the MBC and CPTAC cohorts for example. Those discrepancies can be responsible in part for the differences observed in mutational signatures between the groups.

The fixation step requiring formalin in FFPE samples has long been identified as responsible for generating sequencing artefacts, due to the deamination of cytosine bases, creating abnormal levels of C:G > *T*:A substitutions [17,18]. Recently, Guo et al.*,* demonstrated that the damage caused by formalin could be identified as a formal FFPE signature, and is highly similar to established SBS COSMIC signatures [19]. Indeed, it seems that un-treated FFPE damage signature mimics the SBS30 signature, which is present in over 15 % of the CICLADES samples.

Since no uracil DNA glycosylase (UDG) was used on the sample to remove uracil bases prior to sequencing, unrepaired FFPE stigmas were to be expected and could be the cause for the detection of this SBS30 signature. The absence of this signature in the MSKCC cohort can be due to more extensive filtering of the variants. However, SBS30 is not the only signature linked to FFPE fixation. The use of UDG does not completely prevent the detection of formalin exposure, SBS1 being the reference signature matched with repaired-FFPE damages. This signature was extracted in a third of the METABRIC samples and almost half of the CBSM samples. Since this signature is also associated with aging, its true origin can be guessed by the type of sample used for the extraction. In the METABRIC cohort, fresh frozen tissues were analyzed, eliminating the possibility of repaired FFPE exposure, leaving the more common etiology of aging. However, for the CBSM group, distinguishing between the two origins is not possible since the CPTAC and BROAD samples are fresh frozen tissues, while the MBC samples are FFPE, and no information was available for the SANGER samples. It is also important to consider the size of the populations, which vary drastically from one cohort to another.

The tissue sampling site is also a bias among cohorts and the information available on the subject is not always clear. As stated previously, for the CICLADES, MBC and CPTAC cohorts, we were able to identify the locations of the biopsy, coming from lumpectomy, mastectomy or skin or metastasis in most cases. For the METABRIC cohort, the samples originated from primary breast tumors. When extracting DNA for genomic analysis, sample quality and genetic material yield are primary concerns. Issues can arise when dealing with bone metastasis samples, frequent in BC, as the required process of decalcification can decrease substantially the yield of genetic material [20,21].

The use of Ethylene Diamine Tetra Acetic acid (EDTA) is preferred to reduce this risk [22], but primary tumor samples or soft tissue samples in general remain a better source of quality DNA for genomic analysis. In this context, we can assume that the quality of sequencing of the METABRIC cohort might be higher than that of the CICLADES and CBSM group. However, this bias cannot be further validated as the data regarding sample origin was not available for the rest of the populations.

As stated previously, different methods of sequencing were used between the cohorts. WES covers all coding regions of the genome, while CGP covers a high number of selected coding regions as well as intronic and regulatory regions. Targeted sequencing panels can also include intronic and regulatory regions alongside exons, which is the case for the MSK-IMPACT panel [23], however their coverage can be much smaller than CGPs. Globally, WES is the preferred method for TMB determination, however the design of CGPs and some targeted panels can now also provide accurate estimation of this burden. Here, we evaluated the variations in TMB between cohorts, as well as the number of TMB-high patients in each population. One of the limitations of this comparison is the size of the panel used for the METABRIC cohort, as the minimum of 300 genes covered was not met (panel of 173 genes) [24]. However, we were able to show similar results between the CICLADES and CBSM groups, with no significant difference between their mean number of mutations per Mb (0.69 vs 0.84; *P* = 0.718). This confirms that CGPs can accurately estimate TMB compared to WES on similar populations. The mean number of mutations were significantly higher in the MSKCC group, but this difference is most likely due to the size of the cohort (1203 samples vs 16 and 167 for CICLADES and CBSM, respectively), rather than because of the size of the panel.

Additionally, TMB-high patients (TMB ≥ 10 mut/Mb) were found in all 3 analyzed cohorts: 1, 3 and 35 in the CICLADES, CBSM and MSKCC groups, respectively, amounting to 2.81 % of the compared samples (39 out of 1386 samples). Overall, the TMB results of the CICLADES analysis provides new relevant information to be added to the existing information on the subject.

The method of sequencing also has an impact of the estimation of APOBEC enrichment in samples. Due to the smaller size of the CICLADES cohort, no APOBEC enrichment was found, which was expected as only 16 samples fit the criteria for this analysis and no APOBEC signatures were extracted from the cohort. All analyzed populations had varying distribution of enriched samples, but all had a significantly higher mean number of mutations in the enriched sample groups. This is expected as the APOBEC enzymes are known to play a role in carcinogenesis and can increase sporadically the number of mutations in the genome [25]. The high number of mutations found in both the enriched and not-enriched samples of the CBSM cohort can be explained using WES for sequencing. Indeed, the global coverage of over 40Mb of the CBSM group allow the detection of more mutations than the lower coverage of the targeted sequencing in the MSKCC and METABRIC cohorts.

## Conclusion

We provide here a comprehensive study on genomic signatures published in the literature and the first patients of the CICLADES trial. The use of CGP for sequencing allowed the accurate detection of TMB and relevant signatures, as well as confirming the expected mutation rates of genes of interest in breast cancer, such as *PIK3CA* and *TP53.* Some limitations were found, notably the size of the cohort and the origin of the samples, however, the data provided here remains relevant.

## CRediT authorship contribution statement

**Margaux Betz:** Conceptualization, Data curation, Formal analysis, Investigation, Validation, Writing – original draft, Writing – review & editing. **Andréa Witz:** Validation, Writing – review & editing. **Julie Dardare:** Visualization, Writing – review & editing. **Cassandra Michel:** Visualization. **Vincent Massard:** Investigation, Project administration. **Romain Boidot:** Formal analysis. **Pauline Gilson:** Resources, Supervision. **Jean-Louis Merlin:** Supervision, Validation, Writing – review & editing. **Alexandre Harlé:** Resources, Supervision, Validation, Writing – review & editing.

## Declaration of competing interest

The authors declare that they have no known competing financial interests or personal relationships that could have appeared to influence the work reported in this paper.

## Data Availability

The datasets used and analysed during the current study are available from the corresponding author on reasonable request.
